# Multi Methods for Detecting Natural Toxins

**DOI:** 10.3390/toxins18050204

**Published:** 2026-04-28

**Authors:** Svetlana V. Malysheva

**Affiliations:** Unit Toxins, Service Organic Contaminants and Additives, Scientific Directorate Chemical and Physical Health Risks, Sciensano, Leuvensesteenweg 17, 3080 Tervuren, Belgium; svetlana.malysheva@sciensano.be

Natural toxins constitute a large group of toxic compounds produced by plants, fungi, bacteria and other organisms. While these compounds are typically not poisonous to the producing organisms themselves, their transfer into food, feed, or the environment can result in significant risks to human and animal health. Their chemical diversity, occurrence at low levels, and presence in complex matrices make their reliable detection a persistent analytical challenge. Consequently, continuous advances in analytical methodologies are essential not only for accurate identification and quantification but also for exposure assessment, biomonitoring, and clinical diagnostics.

This Special Issue (SI) of the Toxins journal is dedicated to the theme “Multi Methods for Detecting Natural Toxins”. It provides a collection of articles that emphasise the simultaneous detection of multiple toxins, showcase innovations in sample preparation, and expand the applicability fields of toxin detection across food, feed, and human biomonitoring.

Advances in chromatography coupled to mass spectrometry have significantly enhanced the ability to simultaneously detect toxins of different origins and classes. Malysheva et al. developed and validated a sensitive liquid chromatography-tandem mass spectrometry (LC-MS/MS) method for the simultaneous quantification of hydroxyanthracene derivatives (HADs) in food supplements, addressing the urgent regulatory need for multi-analyte methods following the recent European Commission ban on specific genotoxic HADs [1]. In another study highlighting the chemical diversity of plant toxins, Namdar et al. combined the strengths of LC-MS/MS and gas chromatography-mass spectrometry techniques to establish comprehensive profiles of quinolizidine alkaloids in seeds of wild lupin species, providing essential insights into their complex biosynthesis pathways and potential for crop domestication [2].

Analysis of natural toxins involves handling complex sample matrices; therefore, the development of innovative sample preparation techniques and novel extraction methodologies to overcome the challenges posed by these matrices is highly sought after. Pérez-Álvarez et al. demonstrated the efficacy of magnetic molecularly imprinted polymers for the highly selective and efficient extraction of aflatoxins in complex pig feed matrices, eliminating the need for traditional, more labour-intensive solid-phase extraction [3].

An important highlight of this SI is the transition of analytical methods into clinical and biomonitoring applications. Visintin et al. validated a state-of-the-art ultra-high performance LC-MS/MS method for monitoring *Alternaria* toxins and their phase II conjugates across multiple human matrices, including urine, faeces, and capillary blood. Notably, the use of minimally invasive volumetric absorptive microsampling facilitates large-scale human biomonitoring studies [4]. Similarly, bridging food safety and clinical outcomes, Masquelier et al. applied a targeted LC-MS/MS method to successfully quantify the emetic *Bacillus cereus* toxin, cereulide, in both contaminated food and human faeces during a foodborne outbreak, providing a vital tool for clinical investigations [5].

Many current analytical strategies often cannot differentiate between biologically active and inactive toxins. To close this gap, Li et al. developed a high-throughput neutralising antibody-based cytotoxicity assay capable of precisely identifying and differentiating biologically active ricin and abrin in complex matrices such as milk, coffee, and plasma [6]. In clinical diagnostics, Thilakarathna et al. tackled the issue of discrepancies in results of polymerase chain reaction methods and enzymatic immunoassays for Shiga toxin-producing *Escherichia coli* by demonstrating that mitomycin C can induce Shiga toxin production in clinical isolates, thereby significantly improving diagnostic detection [7]. Highlighting ongoing diagnostic challenges, Prygiel et al. provided a comprehensive review of the evolution of diphtheria toxin detection, emphasising that while the traditional Elek test remains the gold standard, other promising assays (e.g., loop-mediated isothermal amplification), which can potentially be used for rapid point-of-care testing, have been developed [8].

Collectively, the articles of this SI constitute a cohesive scientific output that significantly advances the detection and monitoring of natural toxins. However, as some critical gaps remain, the exploration of novel areas should continue, including the development of standardised reference materials for toxins, the expansion of rapid point-of-care diagnostics, and the refinement of assays that can reliably distinguish between active and inactive toxins. By continuing to innovate in these areas, improvements and advances can be achieved in food and feed control and human biomonitoring, ultimately safeguarding human and animal health against the diverse threats posed by natural toxins.
